# Supplementary material for: Assessment of Foods Associated with Sodium and Potassium Intake in Japanese Youths Using the Brief-Type Self-Administered Diet History Questionnaire
Source: Nutrients. 2021 Jul 9;13(7):2345. doi: 10.3390/nu13072345 (PMC8308755; doi:10.3390/nu13072345)
Supplement: Supplementary file 1 [file nutrients-13-02345-s001.zip › nutrients-1287460-supplementary.pdf]

Supplementary Table S1. Distribution of food intake as medians of quintile strata (g/1000kcal).

	Q1	Q2	Q3	Q4	Q5
Cereals	31.0	45.1	54.4	64.9	80.8
Bread	4.6	11.5	19.5	27.0	38.0
Noodles	10.7	16.6	21.5	28.5	43.5
Vegetables	41.6	64.1	85.3	108.4	151.6
Pickles	0.0	0.7	1.6	4.1	9.4
Fruits	10.6	21.8	34.7	53.2	89.4
Fish	10.5	16.8	22.7	30.5	46.5
Salty fish	0.0	2.4	3.7	5.6	11.5
Meat	17.2	25.2	29.7	35.7	50.2
Processed meat	1.2	2.2	4.3	6.0	10.5
Dairy prodcts	9.1	41.8	65.8	99.8	195.4
Confectionaries	13.5	24.7	35.1	48.5	73.1
Japanese confectionaries	0.0	1.7	2.4	4.0	9.8
Beverages	144.3	260.7	335.7	418.8	576.8
Sugar sweetened beverages	8.4	28.7	59.6	103.1	222.9
Seasonings/condiments	1.1	1.3	1.5	1.8	3.4
Soy and other sauces	14.7	23.4	30.8	40.6	61.3
Noodle soup	0.6	0.7	0.9	1.0	1.2
Soybean paste (*miso)*	41.4	60.6	77.3	92.5	147.7

Stratification was based on grade- and sex-specific quintiles.
